# Coarctation of the aorta and mild to moderate developmental delay in a child with a *de novo *deletion of chromosome 15(q21.1q22.2)

**DOI:** 10.1186/1471-2350-7-8

**Published:** 2006-02-10

**Authors:** Seema R Lalani, Trilochan Sahoo, Merideth E Sanders, Sarika U Peters, Bassem A Bejjani

**Affiliations:** 1 Department of Molecular and Human Genetics, Baylor College of Medicine, Houston, Texas, USA; 2 Department of Pediatrics, Division of Developmental Pediatrics, Baylor College of Medicine, Houston, Texas, USA; 3 Department of Laboratory Medicine, Sacred Heart Medical Center, Spokane, Washington, USA; 4 Health Research and Education Center, Washington Sate University, Spokane, Washington, USA

## Abstract

**Background:**

Deletion of 15q21q22 is a rare chromosomal anomaly. To date, there have been nine reports describing ten individuals with different segmental losses involving 15q21 and 15q22. Many of these individuals have common features of growth retardation, hypotonia and moderate to severe mental retardation. Congenital heart disease has been described in three individuals with interstitial deletion involving this region of chromosome 15.

**Case presentation:**

We report a child with coarctation of the aorta, partial agenesis of corpus callosum and mild to moderate developmental delay, with a *de novo *deletion of 15q21.1q22.2, detected by the array Comparative Genomic Hybridization (CGH). We utilized chromosome 15-specific microarray-based CGH to define the chromosomal breakpoints in this patient.

**Conclusion:**

This is the first description of mapping of an interstitial deletion involving the chromosome 15q21q22 segment using the chromosome 15-specific array-CGH. The report also expands the spectrum of clinical phenotype associated with 15q21q22 deletion.

## Background

Interstitial deletion of chromosome 15q21q22 is an infrequently described chromosomal abnormality. To date, there have been only ten individuals reported, nine with variable deletions involving 15q21 [1-9] and one individual with more distal deletion encompassing the 15q22q25 region [5]. All patients described have moderate to severe mental retardation. The four patients described by Yip *et al*. [1], Fryns *et al*. [2], Martin *et al*. [3] and Liehr *et al*. [4] have comparable cytogenetic breakpoints and share common features, including beaked nose, thin upper lip, and mental retardation. Congenital heart disease was described in one patient with 15q21q24 deletion [5] and two patients with interstitial deletion of chromosome 15q15q21 and 15q15q22 respectively [7,8]. Here, we describe an individual with deletion of 15q21.1q22.2, coarctation of the aorta and mild to moderate developmental delay.

## Case presentation

### Case report

**Figure 1:**
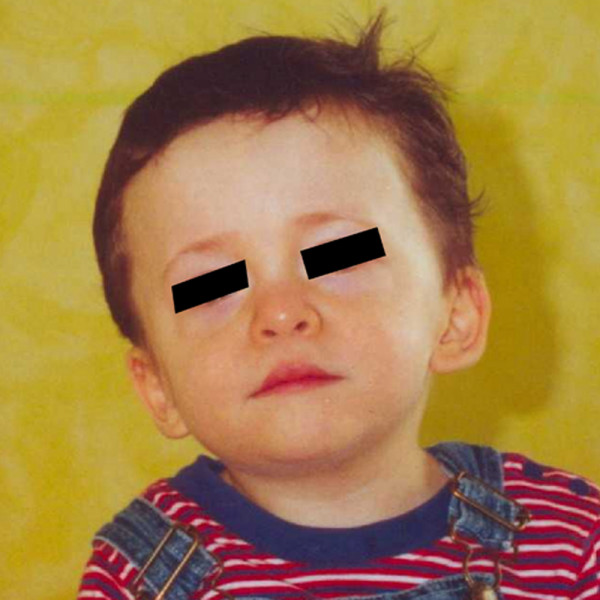
Proband at 20 months of age.

The proband was a 36-week product of a twin pregnancy, born to a 39-year old female. Pregnancy was achieved by *in vitro *fertilization secondary to mother's history of endometriosis. His birth weight was 2.44 kg, (40^th ^percentile), length was 47 cm (50^th ^percentile), and head circumference was 34.5 cm (90^th ^percentile). A cardiac murmur was noted soon after birth. An echocardiogram revealed severe juxtaductal aortic coarctation with near aortic arch interruption. He had a moderately dilated main pulmonary artery and branch arteries, severe septal hypertrophy, moderately depressed biventricular systolic function, biventricular hyprertrophy, and bicommissural aortic valve. He was also noted to have high arched palate, micrognathia, and low set ears with thickened helices. He had fair suck, normal muscle tone and strength. Renal ultrasound showed decreased corticomedullary differentiation, bifid right renal pelvis and moderate pelviectasis. Head ultrasound showed partial agenesis of corpus callosum (see Additional file 1). He underwent surgery for juxtaductal aortic coarctation with end-to-end anastomosis and PDA ligation. At one year of age, his weight was 10.5 kg (50^th ^percentile), length was 77.5 cm (80^th ^percentile) and head circumference was 48.8 cm (95^th ^percentile). He had prominent forehead, midface hypoplasia, thinned lateral eyebrows, intermittent strabismus, low set ears with thickened helices, carp-shaped mouth and retrognathia (Figure 1). He started walking independently at 16 months of age. His renal ultrasound was subsequently repeated and was found to be normal. He had a brief seizure episode at 16 months of age. Since then, he has been treated with Valproic acid and the seizures have not recurred. MRI scan of the brain following the seizure episode showed diffuse delay in myelination and partial agenesis of corpus callosum. EEG showed generalized paroxysmal activity with high voltage slow waves and some short spikes.

At a chronological age of 48 months, he is in a regular classroom with children, a year younger than him. He is able to throw and catch a ball, march to a song, and use a spoon. He is not yet able to walk down stairs using alternating feet. He is able to name body parts, identify some shapes. He speaks in simple phrases, understands prepositions, and can follow simple commands. He receives speech therapy for his speech/language delays. His current level of functioning was evaluated based on teacher's assessment of his development across the domains of gross motor, fine motor, social/emotional, speech/language, and functional skills. Using the Learn and Play questionnaire, his level of functioning was broadly assessed between 23–34 month level, depending upon the specific task, reflecting mild delays in some and moderate delays in other areas. His motor skills are estimated to fall at the 25–26 month-old level, reflecting mild to moderate delays in this area. His language skills are slightly more delayed, falling at approximately the 23–24 month level, reflecting moderate delays. Cognitively, he is able to count from 1–10 in a stable sequence (a 34-month skill).

### Cytogenetics, Comparative genomic hybridization and FISH analyses

**Figure 2:**
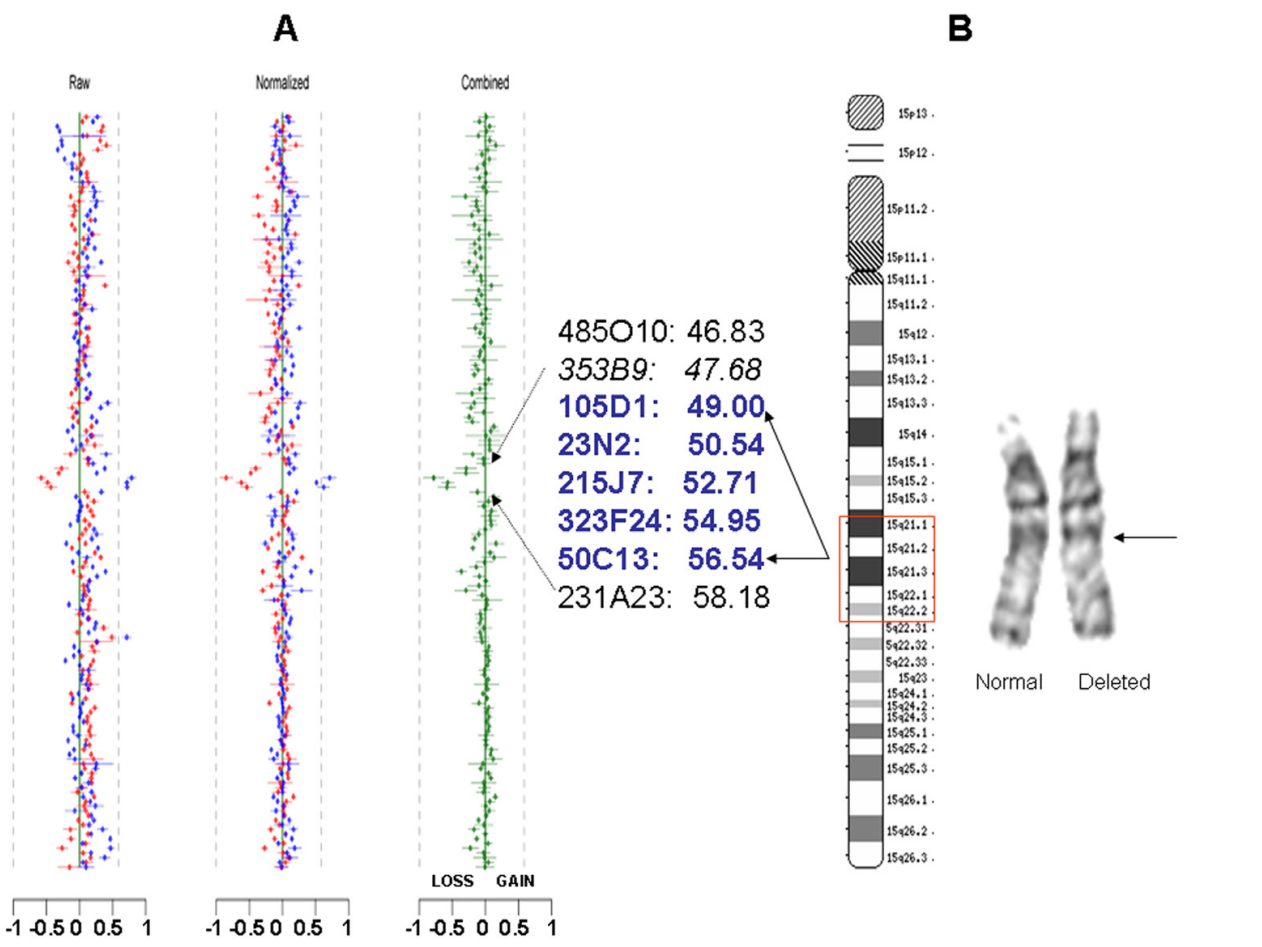
**Chromosome 15 array Comparative genomic hybridization (CGH) analysis and partial karyotype of the case. **(A) The log_2 _ratio plots representing array-CGH analysis using the chromosome 15-BAC microarray. Raw and Normalized data are shown in a clone-by-clone order for both dye-reversal experiments; the combined ratio plot designates loss, gain or normal value for every clone. The ordering of clones (top to bottom) is for chromosome 15 centromere to telomere, followed by all other autosomes and chromosomes X and Y. The overall mean across all clones for the gain state was +0.22, for the loss state -0.34, and for the no-change state -0.04. These values were set as approximate values for predicting a loss or gain for a clone. A loss value is seen for five non-overlapping clones. The clones listed in blue are deleted; the adjacent clones listed in black are not deleted. Clone RP11-353B9 (italicized) was not an arrayed clone but was included for FISH analysis as it was immediately proximal to the deletion boundary. The meagabase position of clones is indicated alongside the clone number. (B) Ideogram of chromosome 15 with demarcation (red box) of the CGH-predicted breakpoints with reference to cytogenetic bands. Partial karyotype, showing normal and deleted chromosome 15, with an apparent interstitial deletion of 15q21.2q22.1.

**Figure 3:**
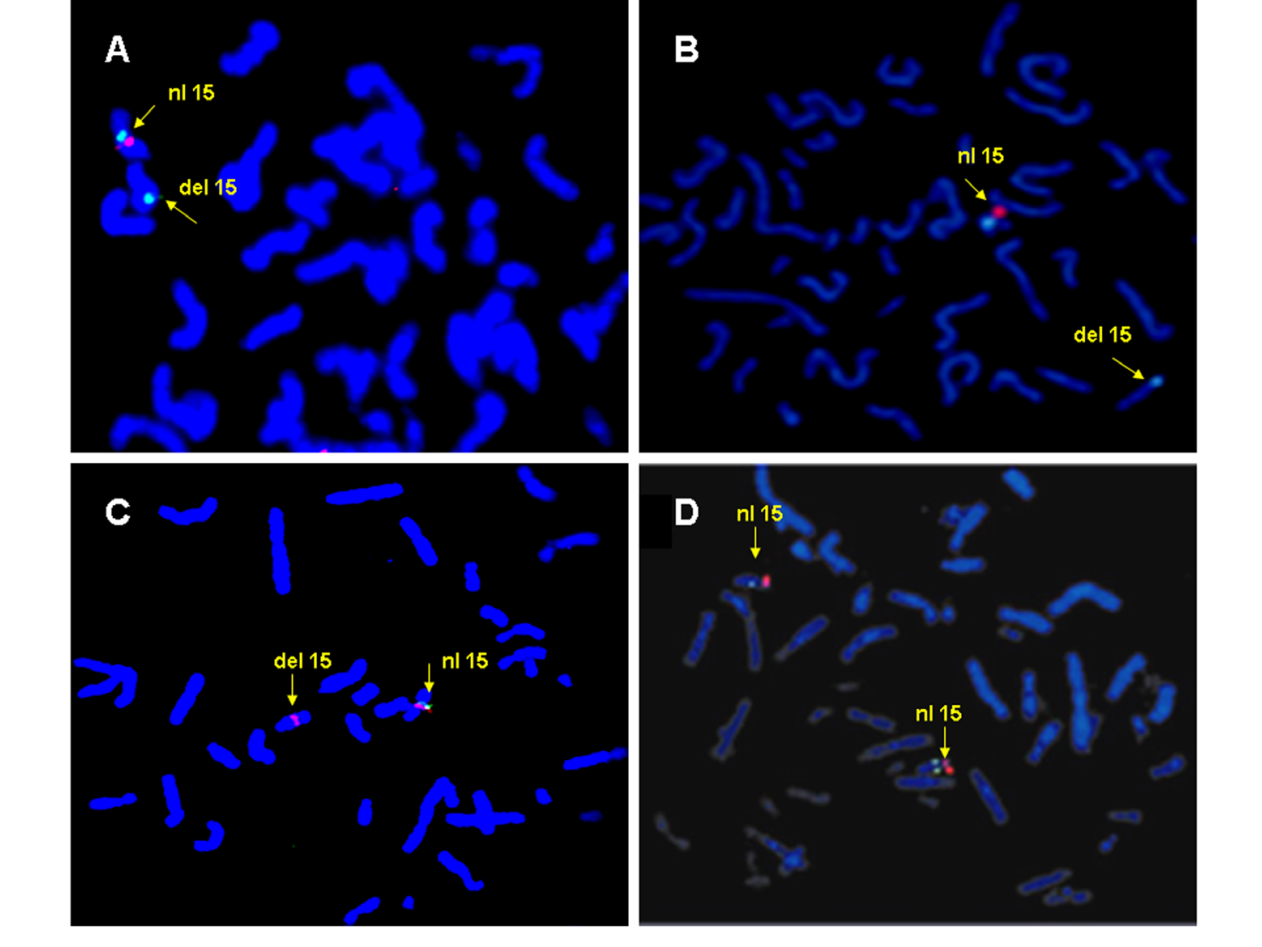
**Fluorescence *in situ *hybridization (FISH) of 15q21.1q22.2 region using BAC probes. **(A) Hybridization with clones RP11-231A23 (green; distal to the deletion interval) and RP11-105D1 (red) showing deletion of one copy of RP11-105D1. (B) Deletion of one copy of RP11-23N2 (red) hybridized with centromeric control probe (green). (C) Hybridization with RP11-50C13 (green) and RP11-485O10 (red; proximal to the deletion boundary) showing deletion of one copy of RP11-50C13. (D) Hybridization with RP11-69G7 (red) encompassing α-tropomyosin gene, shows intact alleles on both chromosomes.

Giemsa-banded chromosome analysis was performed according to standard procedures on peripheral blood lymphocytes. Twenty metaphases were examined at the 550-band level. Further delineation of the size and boundaries of the deletion was carried out by genomic array-based comparative genomic hybridization (CGH) and FISH. For array CGH, we utilized a high-resolution chromosome 15-specific BAC array developed in our laboratory that included 106 BAC clones across 15q. The highest density of clones is across the ~10-Mb 15q11-q14 interval encompassing the common Prader-Willi/Angelman syndrome critical region including the common deletion/duplication breakpoints. Therefore a resolution of greater than 1 clone per megabase for 15q11-q14 and less than 1 clone per megabase for 15q14-qter was achieved in this version of the chromosome 15 array. Additionally the array included 38 clones specific for the subtelomeric region of 41 clinically relevant telomeres for chromosomes 1 to 22, including 12 clones for chromosomes X and 6 clones for Y chromosome. All clones were selected based on physical maps represented in build 34 (2004) of the human genome sequence [10]. Standard protocols for DNA preparation, labeling and hybridization were followed as described previously [11]. A single normal male DNA was used as reference control for analysis of the proband. All array-CGH experiments included dye reversal and two array hybridizations to obtain an accurate ratio [11]. After quantification and normalization of data for each clone on each of the duplicate arrays, the data from dye-reversed pairs were combined, and inferences were made according to a clone-by-clone classification procedure to determine the gain/loss status of each clone for each subject. The clone by clone classification procedure is similar to the independent clone procedure published previously [11]. From a number of test experiments, the overall mean across all clones for the gain state was +0.22, for the loss state -0.34, and for the no-change state -0.04 (Figure 2). These values were set as approximate values for predicting a loss or gain for a clone. BAC clones chosen for FISH analysis were those that mapped within, proximal and distal to the deletion boundaries predicted by the array CGH. These included RP11-485O10 [GenBank: AC090846] and RP11-353B9 [GenBank:AC018927] proximal to and RP11-231A23 [GenBank:AC023190] distal to the deletion interval predicted by CGH (RP11-353B9 was not included in the array but was used for narrowing down the interval by FISH only) and clones RP11-105D1 [GenBank:AC022407], RP11-23N2 [GenBank:AC025917], RP11-215J7 [GenBank:AC009559], RP11-323F24 [GenBank:AC010999] and RP11-50C13 [GenBank:AC018904] within the interval (Figures 2 and 3). Additionally, FISH with RP11-69G7 [GenBank:AC087612] encompassing α-tropomyosin gene was carried out prior to the array-CGH analysis as this was an important candidate gene for the cardiac phenotype in the proband and was located within the broader interval as estimated by G-banding and karyotype. FISH analyses were carried out as previously described [12]. At least ten metaphase preparations were scored for each hybridization.

## Results

G-band analysis of a peripheral blood sample revealed an abnormal male karyotype: 46, XY, del(15)(q21.2q22.1). Parental chromosomes were normal. Deletion at 22q11 (DiGeorge syndrome critical region) was excluded by FISH. Array CGH revealed an interstitial deletion with loss of 5 non-overlapping clones across 15q21.1-q22.2 (RP11-105D1-> RP11-50C13; Chr15q- 49.00 Mb to 56.54 Mb). The proximal boundary was telomeric to clone RP11-353B9 and distal boundary was centromeric to RP11-231A23. This included the interval between 15q21.1 to 15q22.2 and spanned a genomic segment of at least 7.54 Mb (Figure 2). FISH analysis using clones within and flanking the boundary confirmed the size and extent of the deletion (Figure 3). All of the five clones predicted by CGH to be within the deleted interval were deleted from one copy of the patient's chromosome 15. Additional FISH with RP11-69G7 that contains *TPM1*, the α-tropomyosin gene showed two hybridization signals, confirming the presence of both copies of the gene in this patient.

## Discussion

Deletion of 15q21 is an infrequently described chromosomal abnormality. Of the ten reported cases, many have common features of beaked nose, hypoplastic alae nasi, thin upper lip, truncal obesity, growth retardation, hypotonia and moderate to severe mental retardation [[1-3]] (see Additional file 1). Our patient's phenotype included prominent forehead, midface hypoplasia, intermittent strabismus, low set ears, carp-shaped mouth, retrognathia, coarctation of the aorta, partial agenesis of corpus callosum, and mild to moderate developmental delay. Two patients with interstitial deletion of 15q15q21 have also been described with craniosynostosis [7,9]. Neurological problems including hypotonia and seizures are also seen in many individuals with the deletion of this region of chromosome 15q. Congenital heart defect with septal hypertrophy and dilatation of the aorta and pulmonary artery was described in one patient with the deletion of 15q21q24 [5], who died at 8 months of age. Another patient in the same report had deletion of 15q22q25 and frequent cyanosis of the extremities with no heart murmur, and died at 2 years of age with severe respiratory illness. Five other patients with comparable interstitial deletion [[1-4,6] had no evidence of congenital heart disease. Two additional cases with interstitial deletion of chromosome15q15-21 have been described with atrial and ventricular septal defects and Tetralogy of Fallot with septal hypertrophy, respectively [7,8]. We explored probable hemizygous deletion of a cardiac specific gene within the 15q21.1-q22.2 interval responsible for structural heart defects in these individuals. Within this deletion interval, there are few genes that are known to be expressed in heart, including *ARPP-19 *[13], *RAB27A *[14], and *ADAM10 *[15]. The α-tropomyosin gene, *TPM1 *maps to 15q22.2 and is located within the broader chromosome 15 deletion interval, as suggested by the G-band analysis. Heterozygous point mutations in *TPM1*, account for <5% cases of familial hypertrophic cardiomyopathy [16]. The phenotype ranges from a benign course to severe hypertrophy with progression to dilated cardiomyopathy [16,17]. In view of our patient's cardiac phenotype, which included septal hypertrophy and juxtaductal aortic coarctation, *TPM1 *appeared a good candidate gene to investigate if it was included within the deleted region. Prior to performing the array-CGH, we carried out FISH with RP11-69G7 encompassing the *TPM1 *gene (Figure 3). We established that *TPM1 *was not included within the deletion interval and ruled out the deletion of *TPM1 *gene causing left ventricular outflow tract obstruction observed in this patient. The array-CGH subsequently confirmed that RP11-69G7 maps approximately 4 Mb distal to RP11-50C13, the most telomeric clone deleted on the array-CGH in our patient.

## Conclusion

The array CGH refined the interstitial deletion in our patient to 15q21.1-q22.2. Microarray-based Comparative genomic hybridization is a powerful method to detect and analyze genomic imbalances. Array CGH using large insert clones is a very useful tool for detecting microdeletions or duplications that are well below the level of detection on high resolution banded karyotype analysis. We have used chromosome 15-specific microarray to define the 15q breakpoints in this case, thus providing a better opportunity for genotype/phenotype correlations in other similarly affected individuals.

## Competing interests

The author(s) declare that they have no competing interests.

## Authors' contributions

SRL carried out the clinical evaluation, the FISH analyses and drafted the manuscript. TS performed the CGH analysis and MES carried out the FISH analyses. SUP assisted in interpretation of the developmental assessment of the patient. BAB participated in the clinical assessment of the patient, conceived the study design and coordinated the study progress. All authors read and approved the final manuscript.

## Pre-publication history

The pre-publication history for this paper can be accessed here:



## Supplementary Material

Additional File 1 **Table: Clinical manifestations in individuals with deletion encompassing 15q15q22 region **Clinical overview of all the known individuals with 15q15q22 region deletion. Click here for file
