# Supplementary material for: Coarctation of the aorta and mild to moderate developmental delay in a child with a de novo deletion of chromosome 15(q21.1q22.2)
Source: BMC Med Genet. 2006 Feb 10;7:8. doi: 10.1186/1471-2350-7-8 (PMC1397801; doi:10.1186/1471-2350-7-8)
Supplement: Additional File 1 — Table: Clinical manifestations in individuals with deletion encompassing 15q15q22 region Clinical overview of all the known individuals with 15q15q22 region deletion. [file 1471-2350-7-8-S1.doc]

## **Additional file 1: Clinical manifestations in individuals with deletion encompassing 15q15q22 region**

| **References** | **Karyotype** | **Developmental**  **delay** | **Failure to thrive** | **Hypoplastic alae nasae** | **Micrognathia** | **Thin upper lip** | **High arched palate** | **Eyes** | **Ears** | **Heart** | **Urogenital** | **CNS** | **Brain imaging** |
| --- | --- | --- | --- | --- | --- | --- | --- | --- | --- | --- | --- | --- | --- |
| Present report | 46,XY,del(15)(q21.1q22.2) | Mild-  Moderate | - | - | + | - | + | Strabismus | Thickened helices | CoA,  septal hypertrophy  BAV | Hydronephrosis | Single episode of seizure | Partial ACC |
| Martin [3] | 46,XY,del(15)(q21.2q22.1) | Moderate | - | - | - | + | NR | Strabismus | NR | NR | Normal | Hypotonia | NR |
| Liehr [4] | 46,XX,del(15)(q21.1q21.3) | Moderate | - | + | NR | + | NR | NR | Low set | Normal | Normal | Hypotonia, normal EEG | Normal |
| Yip [1] | 46,XY,del(15)(q21.1q22.1) | Moderate | - | + | + | + | NR | Myopia, strabismus | Low set | NR | Cryptorchidism | Hypotonia | NR |
| Fryns [2] | 46,XY,del(15)(q21) | Severe | + | + | NR | + | NR | Hypopigmented iridis, microcornea | Low set, posteriorly rotated | NR | Genital  hypoplasia | Spastic paraplegia,  microcephaly | NR |
| Pramparo [6] | 46,XY,del(15)(q21) | NR | + | NR | + | NR | NR | Blepharophimosis | Low set, large | PFO | Normal | Hypotonia | NR |
| Formiga [5] | 46,XX,del(15)(q22q25)  (Case 1) | Severe | + | + | + | - | + | Microphthalmia, hypopigmented irides | Large with thickened helices | NR | Normal | Hypertonia,  microcepaly, seizures | Cortical  atrophy |
|  | 46,XX,del(15)(q21q24)  (Case 2) | Severe | - | - | + | - | + | Microphthalmia,  coloboma of iris, hypopigmented iridis | Large with poorly defined helices | Septal hypertrophy | NR | Hypotonia, abnormal EEG | NR |
| Fukushima [9] | 46,XY,del(15)(q15q22.1) | Severe | + | + | + | NR | NR | NR | Low set and large | NR | Cryptorchidism | Hypotonia | NR |
| Koivisto [7] | 46,XY,del(15)(q15.2q21.2) | Severe | + | + | + | NR | + | Ptosis | Left ear hypoplasia | ASD,  VSD | Cryptorchidism | Seizures, microcephaly | DLV,  HCC |
| Shur [8] | 46,XX,del(15)(q15q22.1) | Severe | - | + | + | + | + | NR | Cupped  hypoplastic superior helix | TOF,  septal  hypertrophy | Bilateral hydronephrosis | Hypotonia | DLV |

NR= not reported, CoA=coarctation of the aorta, BAV=bicommissural aortic valve, ASD=atrial septal defect, VSD=ventricular septal defect, TOF=Tetralogy of Fallot, PFO=patent fossa ovalis, ACC=agenesis of corpus callosum, DLV=dilated lateral ventricles, HCC=hypoplastic corpus callosum
